# Guidelines for short-term medical missions: perspectives from host countries

**DOI:** 10.1186/s12992-022-00815-7

**Published:** 2022-02-19

**Authors:** Patti Tracey, Ethan Rajaratnam, Julie Varughese, David Venegas, Belinda Gombachika, Mercy Pindani, Elizabeth Ashbourne, Alexandra Martiniuk

**Affiliations:** 1 Trent Fleming School of Nursing, Trent University, 1600 West Bank Dr, Peterborough, ON K9L 0G2 Canada; 2 Department of Epidemiology and Biostatistics, Schulich School of Medicine & Dentistry, Western University, London, ON Canada; 3 Americares, 88 Hamilton Ave, Stamford, CT USA; 4 Trent Fleming School of Nursing, Trent University, Tegucigalpa, Honduras; 5 Kamuzu College of Nursing, University of Malawi, P.O. Box 415, Blantyre, Malawi; 6 Kamuzu College of Nursing, University of Malawi, Private Bag 1, Lilongwe, Malawi; 7 Partnership for Quality Medical Donations, 326 First Street, Ste 32, Annapolis, MD 21403 USA; 8 Faculty of Medicine and Health, The University of Sydney School of Public Health, Edward Ford Building A27, Sydney, NSW 2006 Australia; 9 The George Institute for Global Health | Office of the Chief Scientist, Sydney, Australia; 10 The University of Toronto | Dalla Lana School of Public Health, Toronto, Canada

**Keywords:** Short-term medical missions, Guidelines, Best practices, Quality improvement, Low-and middle-income countries, Decolonize Global Health

## Abstract

**Background:**

In the past decade, there has been increasing guideline development for short-term medical missions (STMMs) traveling from high-income to low- and middle-income countries for the purpose of supporting health care services. The ethics of STMMs is criticized in the literature and there is frequently a lack of host country collaboration. This typically results in guidelines which are developed through the lens of the sending (high-income) countries’ staff and organizations. The aim of this paper is to evaluate an existing best practice guideline document from the perspective of host country participants with knowledge of STMMs from Honduras, Malawi, and the Philippines.

**Methods:**

The guideline used for the evaluation consisted of nine best practice elements that were discerned based on literature and the experience of those working within the field. Semi-structured interviews were conducted in a cross-sectional study with participants (*n* = 118) from the host countries. Thematic analysis was conducted by two researchers and the results were assessed by working group members to confirm interpretations of the data.

**Results:**

Overall, participants expressed a strong interest in having more structured guidance surrounding STMM practices. There was a positive response to and general acceptance of the proposed STMM guidelines, although participants found the 24-page document onerous to use; a companion checklist was developed. The key themes that emerged from the interviews included collaboration and coordination, care for hard-to-reach communities, capacity building, critical products and essential medical supplies, and opportunity and feasibility.

**Conclusions:**

Host input suggests that the guidelines provide structured regulation and coordination of the medical mission process and have the potential to improve the way STMMs are carried out. The guidelines have also proven to be a useful tool for the actual implementation of STMMs and can be a tool to strengthen links and trust between mission teams and local health staff. However, local contexts vary considerably, and guidelines must be adapted for local use. It is recommended that STMM teams work in conjunction with host partners to ensure they meet local needs, increase capacity development of local health workers, and provide continuity of care for patients into the local system.

**Supplementary Information:**

The online version contains supplementary material available at 10.1186/s12992-022-00815-7.

## Abstract (es)

**Antecedentes:**

En la última década, ha habido un incremento en el desarrollo de guías para las misiones médicas de corto plazo (STMM) que viajan desde países de ingresos altos a países en vías de desarrollo con el fin de apoyar los servicios de atención médica. La ética de las (STMM) es criticada en la literatura y hay una falta frecuente de colaboración entre los países anfitriones. Esto normalmente da como resultado directrices que se desarrollan a través de la lente del personal y las organizaciones de los países que envían (países de altos ingresos). El objetivo de este documento es evaluar un documento guía de mejores prácticas existente desde la perspectiva de los participantes del país anfitrión con conocimiento de las misiones médicas de corto plazo (STMM) de Honduras, Malawi y Filipinas.

**Métodos:**

la directriz utilizada para la evaluación consistió en nueve elementos de mejores prácticas que se discernieron en base a la literatura y la experiencia de quienes trabajan en el campo. Se realizaron entrevistas semiestructuradas en un estudio transversal con participantes (*n* = 118) de los países anfitriones. El análisis temático fue realizado por dos investigadores y los resultados fueron evaluados por miembros del grupo de trabajo para confirmar las interpretaciones de los datos.

**Resultados:**

En general, los participantes expresaron un gran interés en tener una guía más estructurada en torno a las prácticas de las misiones médicas de corto plazo (STMM). Hubo una respuesta positiva y una aceptación general de las pautas de las misiones médicas de corto plazo (STMM) propuestos, aunque los participantes encontraron oneroso el uso del documento de 24 páginas y se desarrolló una lista de verificación complementaria. Los temas clave que surgieron de las entrevistas incluyeron colaboración y coordinación, atención a comunidades de difícil acceso, desarrollo de capacidades, productos críticos y suministros médicos esenciales, y oportunidad y viabilidad.

**Conclusiones:**

Los comentarios del anfitrión sugieren que las directrices proporcionan una regulación y coordinación estructuradas del proceso de la misión médica y tienen el potencial de mejorar la forma en que se llevan a cabo las misiones médicas de corto plazo (STMM). Las pautas también han demostrado ser una herramienta útil para la implementación real de de las misiones médicas de corto plazo (STMM) y pueden servir para fortalecer los vínculos y la confianza entre los equipos de misión y los sistemas de salud locales. Sin embargo, los contextos locales varían considerablemente y las pautas deben adaptarse para el uso local. Se recomienda que los equipos de las misiones médicas de corto plazo (STMM) trabajen en conjunto con los socios anfitriones para garantizar que satisfagan las necesidades locales, aumenten el desarrollo de la capacidad de los trabajadores de salud locales y brinden continuidad de atención a los pacientes en el sistema local.

## Background

Prior to the COVID-19 pandemic, there was substantial interest from healthcare professionals and trainees in clinical and public health programs from high-income countries (HICs) in short-term medical missions (STMMs) to provide low- and middle-income countries (LMICs) with health care, health promotion, health prevention activities, or some combination of supports [1–5]. For instance, between 800,000 and 1,100,000 individuals in the United States reported volunteering internationally, including in STMMs, each year from 2004 to 2012 [6]. As defined by Martiniuk et al. [1], a STMM is “a short trip of 1 day to 2 years by a healthcare professional to a LMIC to provide direct medical care to the population at large, or to a particular subset of the population identified by their particular health need, age group, or cultural group”.

The overall effectiveness of STMMs is often questioned, however, as the goals of sending organizations and host communities are often incongruent [7, 8]. They are also criticized since they are often not integrated in the local healthcare system, they usually do not provide continuity of care to patients, and their quality of partnership and care can vary greatly between different medical missions [9]. The literature raises further important questions regarding the overall value, long-term impact, and potential unintended consequences of STMMs, along with their often uncoordinated and unregulated nature [2, 8, 10–12]. One of the most troubling concerns with STMMs is their lack of mutually beneficial and sustainable partnerships between sending organizations and host communities. This often creates an overlap in services provided to the community, which may serve to undermine or overburden the existing local health care system [13–15]. This issue is further exacerbated by cultural insensitivities and by the perceptions held by both host and sending participants that foreign health care practitioners and practices are superior to those of the host country [11, 16–18]. An additional issue is that, as stated by Esposito et al. [19], there is “limited oversight of the care provided by such missions… no government agencies or accrediting bodies currently exist to assess the scope and impact of STMM care or to establish standards of care for such missions”.

STMM guidelines are emerging and could be important tools to monitor, evaluate and ultimately improve the conduct and impact of STMMs [20]. Although the terms ‘guidelines’ and ‘standards’ are frequently used interchangeably in the literature [4, 21], ‘guidelines’ refer to “statements that include recommendations… that are informed by a systematic review of evidence and an assessment of the benefits and harms of alternative care options”, as defined by the Institute of Medicine, and is thus a more suitable term, therefore it was used consistently throughout this study and article [22]. STMM guidelines have been gaining global popularity over the past decade, but there are questions surrounding the quality of these guidelines [20]. Host country representation and shared perspectives within these guidelines are inconsistent or lacking, as shown by Lasker et al. [5], who found that the majority of the 27 STMM guidelines they identified in the literature only represented the ideas and perspectives of participants from HICs, rather than those from host country participants [11]. Host stakeholders are often not consulted in the creation or evaluation of the guidelines, despite their input being recognized by most as an essential component of this process [4, 5, 7, 11, 14, 16, 23–27]. Host communities are capable of assessing their own needs, consequently the exclusion of such perspectives further serves to undermine the partnership between sending organizations and receiving communities [4, 13, 25, 28]. This disregard for host perspectives is promotive of neo-colonial values, since it serves to perpetuate power differentials between sending and host stakeholders [8, 16, 18, 25, 29, 30]. There is a growing movement to decolonise global health and Khan et al. [31] stressed the need for emphasis on challenging “ingrained systems of dominance and power in global health work to improve the health of populations and limit inequities whether this occurs between countries, or within countries”. As recommended by Roche et al. [4], “clear guidelines are needed to create global standards to ensure that the services delivered are beneficial not only to patients, but also more broadly to the healthcare systems of recipient countries”. The quality of guidelines is not their only issue. Equally problematic is that few attempts have been made to implement the guidelines in practice, and the impact that those few implemented guidelines have had on engagement, partnership, and clinical care have then rarely been evaluated [32].

Our work aims to address the real-world problem that for many people, particularly the poor and marginalised in LMICs there is little to no access to health care. To address this gap in health services, for decades health professionals from high income countries have been travelling to LMICs to volunteer their professional skills for short periods of time (STMMs). The quality of partnerships and care provided by these STMMs is variable and there have been calls to improve their quality and collaboration. In the literature STMM guidelines have been developed but these do not include the input of people living in LMICs. Hence our study was initiated to analyze existing guidelines for STMMs and to gather the views of those in LMICs. Our study contributes to the crucial movement to decolonize global health.

Thus, there was a need to draft a code of practice creating guidelines that better integrate host country perspectives and are representative of both HICs and LMICs. This paper aims to share the findings of a cross-sectoral, quality improvement study evaluating best practices for STMMs in Honduras, Malawi, and the Philippines, which focused on examining and strengthening the manner in which these medical missions are conducted through the lens of the host country stakeholders who receive volunteer healthcare workers. This study is part of the Partnership for Quality Medical Donations Health System Strengthening/Medical Mission’s (PQMD HSS/MM) multi-year initiative to explore the shortcomings of current medical mission practices and use of guidelines, and to design and develop guidelines that better connect medical mission volunteers with the needs and priorities of host countries’ healthcare systems.

## Methods

The initial draft guidelines were developed from a literature review, and gap analysis of existing STMM codes of practice such as standards, or overarching principles. Through multiple consultative phases, the working group members (Additional file 1) from Canada, USA and Honduras selected and developed nine proposed guidelines from those currently in practice (or not in practice) [4, 5, 10, 21, 33]. The aim was to create guidance through the development of common best practices designed to better align sending organizations from HICs and host organizations from LMICs, and to focus on developing sustainable partnerships, which are a key element of guidelines [34]. The nine best practices (within the guidelines document (Additional file 2)) outline the principles for supporting partnerships and mutual respect in the pre-departure phase, during in-country implementation, and in the post-STMM follow-up. These core elements also focus on improving the process and practice of medical missions and programs, as well as on training and capacity building efforts. They are categorized as: assessment, partnership/alliance, governance, code of conduct, preparation, implementation, training and capacity building, sustainability, and monitoring and evaluation (Table 1). The guidelines document (Additional file 2) was finalized in 2019 following the three-country evaluation by host participants and is reviewed annually by the PQMD guidelines committee [9].

**Table 1:** The best practice guidelines recommended by the PQMD, associated with their corresponding chronological stage of the STMM process

Stage	Guidelines
Pre-departure	1 - Assessment
	2 - Partnership and Alliance
	3 - Governance
	4 - Code of Conduct
	5 - Preparation
In-country	6 - Implementation
	7 - Training and Capacity Building
Post medical mission follow-up	8 - Sustainability
	9 - Monitoring and Evaluation

### Study design

This study used a cross-sectional evaluation design to review the draft set of nine best practice guidelines as categorized by the PQMD/HSS-MM’s working group members. They were written in English and translated into Spanish before the evaluation.

### Study setting: country selection

Honduras, Malawi, and the Philippines were selected as the initial sites for stakeholder research given the high volume of STMMs in these countries, including the high numbers of both foreign and domestic STMMs (i.e., in the Philippines). These countries receive many foreign STMMs from the top four sending countries (Canada, Australia, the United States of America, and the United Kingdom). The literature indicates that most health professionals participating in STMMs are from the United States, while Honduras is the most frequent host country for medical missions [1, 3–5, 32].

These countries were also selected for guideline development and evaluation since they have complex differences between them in terms of culture, geography, language, and health systems and services. As well, they are each in different global regions (Central/South America, African continent, Asia Pacific). Receiving countries vary substantially across these factors, which creates a major challenge for the standardization of STMM guidelines and in the development of sustainable partnerships between host and sending countries, making it essential to examine the PQMD HSS/MM guidelines across multiple types of receiving countries. Overviews of the Honduran, Malawian, and Filipino healthcare contexts are provided next, and Table 2 outlines their demographics and key health outcome metrics for comparison.

**Table 2:** Host country demographics and key health metrics

	Honduras	Malawi	Philippines
Population (in millions)	9.113^a^	18.092^b^	103.320^c^
Life expectancy (in years)	73 (males)^d^ 78 (females)^a^	61 (males)^b^ 67 (females)^b^	66 (males)^c^ 73 (females)^c^
GDP ($)	4270^a^	750^b^	7820^c^
% of GDP spent on health	8.7^a^	11.4^b^	4.7^c^
Maternal mortality (per 100,000)	65^d^	349^e^	121^f^
Mortality in children less than 1 year of age (deaths per 1000 live births)	24^g^	66^h^	19.9^i^
Mortality in children under-5 years of age (deaths per 1000 live births)	18^a^	50^b^	28^c^

^a^https://www.who.int/countries/hnd/en/

^b^https://www.who.int/countries/mwi/en/

^c^https://www.who.int/countries/phl/en/

^d^https://www.who.int/gho/maternal_health/countries/hnd.pdf?ua=1

^e^https://www.who.int/gho/maternal_health/countries/mwi.pdf?ua=1

^f^https://www.who.int/gho/maternal_health/countries/phl.pdf?ua=1

^g^https://www.paho.org/salud-en-las-americas-2017/?p=4280

^h^https://www.afro.who.int/sites/default/files/2017-05/Malawi-ccsbrief-en.pdf

^i^http://www.healthdata.org/philippines

#### Honduras

Honduras is a republic of Central America whose population is divided into 18 separate regions (known as departments), which collectively include 298 municipalities, 3731 villages, and 30,591 hamlets [35]. Honduras’ healthcare system is a complex mix of public, private (for-profit and not-for-profit), and NGO-provided services. The health sector consists of a public subsector made up of the Ministry of Health (Secretaría de Salud), which plays the directory and regulatory role for the sector, and the Honduran Social Security Institute (El Instituto Hondureño de Seguridad Social), which is responsible for collecting and managing fiscal resources and the required contributions made by workers and employers.

#### The Philippines

The Philippines is a Southeast Asian island nation located in the western Pacific Ocean. It includes dozens, if not hundreds, of unique Indigenous populations, and its citizens are spread across thousands of islands [36]. The health system is decentralized, offers universal health coverage reaching 92% of the population [36], and sees steadily improving health indicators. Despite this, it still lags behind the benchmark-comparator countries, as the Philippines is regularly affected by typhoons, earthquakes, and volcanoes. As well, armed unrest in parts of the country (notably the south) have displaced hundreds of thousands of people, creating further hurdles for the health system [37].

#### Malawi

Malawi is a landlocked country in southeastern Africa that is divided into three regions - northern, central, and southern. The country has 28 districts, which are divided into traditional authorities ruled by chiefs. These traditional authorities are further subdivided into villages, which form the smaller administrative units. Approximately 84% of the population live in rural areas, while the remaining 16% live in urban centers [38]. Health services in the public sector are struggling to an extreme degree, as the Ministry of Health (2017) reported a 45% vacancy rate of clinical staff within the public sector, so private for-profit and private not-for-profit health services are especially essential [38]. The Christian Health Association of Malawi is one of the most notable private not-for-profit organizations in the country, since it provides approximately 30% of Malawian healthcare services [39].

### Study sample

Participants included healthcare providers; individuals from universities and professional affiliates, local NGOs, international NGOs, and faith-based NGOs; and local regional and Ministry of Health officials. We sampled from three or more regions in each country to ensure a mix of urban versus rural respondents. The interviewees were enrolled using purposive sampling by region and organization type, followed by snowball sampling. Interviewees were enrolled and data collected until saturation was reached, within Honduras and Malawi. In the Philippines, the sample was limited by time, with only one in-country research trip and saturation was not met. Data were collected in person through face-to-face interviews between November 2017 and May 2018.

Ninety-five percent of the participants were living and working full-time in their respective countries. Table 3 illustrates the distribution of participants (*n* = 118) by host country and grouping, with numbers specified for those who assessed proposed guidelines, and how many were interviewed. Ethical approval was obtained (Trent University REB#25339). The initiative was voluntary, and the interviews were anonymous. Consent was obtained and semi structured interviews conducted in a private, quiet location chosen by the participants. Interviews lasted from 30 to 45 min. Introductory email exchanges were initiated 1 to 2 weeks before the in-country research trip, and the guidelines document was shared electronically for review prior to the interview date.

**Table 3:** Distribution of stakeholder participants (*n* = 118) by host country and stakeholder type

Stakeholder Group	Malawi	Philippines	Honduras	Total
Professional training organizations, universities, and health councils	4	4	4	12
Local healthcare providers				56
Doctors and nurses	9	4	36	
Community leaders	1	0	6	
International healthcare providers working locally in host countries for more than 12 months	4	1	4	9
International healthcare providers living in Canada or the United States and traveling biannually to host countries for STMMs	2	5	10	17
Government officials^a^	5	1	6	12
International NGOs	0	5	7	12
**Total**	25	20	73	**118**

^a^Government officials and regional representatives

Semi-structured interview questions were modified to suit the stakeholder type but gathered the same information regarding the individual or the organization that they represented. Interviews explored personal experiences, perspectives, and challenges and examined current practices or established processes (or both) in the individual’s country of residence as they related to medical missions.

The participants from Malawi and the Philippines were English-speaking, and the interviews were conducted in English. In Honduras, a translator who had experience working in rural regions and an understanding of different cultural practices facilitated the interviews. All participants were adults over the age of 18 years. Patients as recipients of STMMs were not included in this study.

### Data analysis

The two researchers (PT and JM) independently reviewed the field notes and transcripts to identify topics for thematic analysis [40]. Key ideas were identified, coded, and categorized to respond to the evaluation and its nine different guidelines. The researchers met on multiple occasions to discuss and to reach a consensus on the patterns, themes, and analysis threads that emerged. Four themes were identified and shared with the working group members to confirm the interpretations of the investigators, and then compared and contrasted with the members’ and investigators’ existing experiences with medical missions and the current literature.

## Results

A total of 118 interviews were conducted in both urban and rural settings across the three countries using semi-structured interview prompts, which included a review of the nine PQMD best practice guidelines (Table 1). In Honduras, 73 stakeholder participant interviews and one focus group were conducted in four regions: Francisco Morazán, Yoro, Cortez (Pena Blanca), and Valle. In Malawi, 25 stakeholder participant interviews and one focus group were conducted in the three regions: North, Central, and South Malawi. In the Philippines, 20 stakeholder participant interviews were conducted in Metro Manila, the Leyte province, and the Iloilo province.

The key themes that emerged from these interviews included collaboration and coordination, cultural context, critical products and essential medical supplies, and opportunity and feasibility (including capacity building), along with overall guideline recommendations.

### Collaboration and coordination

Overall, participants expressed a strong interest in having more structured guidance surrounding STMM practices. One participant from Malawi indicated, *“most teams are collaborative however, perhaps they lack guidelines”* and another from Honduras commented, *“brigades (STMMs) are important if they have a focus and support local healthcare, government and our community programs”.* There was overall agreement that all volunteers should strive to ethically and optimally contribute to locally led efforts. A general consensus from all three countries was that STMMs should support existing local healthcare, government, and community programs.

There is a need to identify the minimum requirements for culturally accepted best practices [41]. Medical missions to Malawi differ from others in that *teams may be very small and may even consist of only one or two individuals*. Consistently, participants from all three countries reported that there is a need for overall coordination and collaboration across all types of missions, whether those missions involve large teams or individuals. Improved coordination prior to medical mission trips is needed and will naturally help to promote more collaboration through the entire process.

Host interviewees recommend that, prior to departing for a STMM, all members of the STMM undertake a short open-access course regarding best-practices for STMMs, as well as at least a short course in local language and culture. Most importantly, the members of the STMM should partner with a local health professional(s) to ensure that the STMM meets local needs and provides continuity of care for the local system, and also supports capacity development of local health workers.

### Cultural context

Cultural issues surrounding STMMs were frequently brought up during the interviews by the stakeholder interviewees. One participant in Malawi stated that “*teams are arriving without a full understanding of the local context, including, for example, cultural differences, local disease prevalence—which includes rising rates of non-communicable diseases—and pertinent social issues, including gender-based violence*.”

It was strongly recommended by participants from all three countries in the study that STMM volunteers adhere to local licensing and legal requirements [41]. Participants acknowledged that there is a wide array of in-country requirements for medical and nursing licensing. In some instances, licensing is a very involved and lengthy process and includes paying a fee to the health councils of a host country. In other cases, simply carrying your medical or nursing license from your home country and voluntarily registering your STMM with the local Ministry of Health is sufficient for licensing purposes. In Honduras, there is currently no licensing or guidelines for medical missions. In Malawi, health councils are used for the registration of foreign healthcare providers, but there are no guidelines for teams or the donation of supplies. In the Philippines, there are both formal licensing and established guidelines for medical missions and all medical donations [42].

Photography is a particularly sensitive topic for team volunteers. On the negative side of things, these photos may be used to illustrate the “*worst of the worst*” in terms of living conditions or medical conditions in a host country when the health professional returns home. The photos can also be used by volunteers to demonstrate their experience. There are appropriate uses of photos however, for instance, a photo, taken with consent of all those involved with the medical mission trip, not simply the health professionals providing services. Or a photo taken for medical education, with permission of the patient. In fact, these issues were raised and a participant from Honduras suggested *“that there should be a separate code of conduct for photos taken by visiting health professionals.”*

### Critical products and essential medical supplies

Pharmaceuticals are a critically needed resource and the acquisition of medications is a driving force for engagement with foreign medical missions. Consistently, participants discussed the disconnect between the identified local needs and government-level supplies, available products, and medicine procurement, especially for rural and hard-to-reach clinics. In both Malawi and Honduras, multiple participants asserted that “*quality of care”* is routinely compromised because of a consistent lack of medicines and medical supplies. An exemplar illustrates this burden, whereby a doctor in Malawi who appeared exhausted and frustrated before his shift even began said, *“I am working in the Diabetes clinic today and have no Metformin (hypoglycemic medicine) for the patients”*. Medical mission teams may help support product needs; however, as a doctor in Honduras shared, there are differences in local formularies and in overall familiarity with medicines that need to be considered when foreign medical missions carry medications to prescribe during short-term medical service visits in other countries. As well, donating medications and medical supplies can affect a region’s (or a country’s) logistics and supply chain with inconsistent influxes of resources affecting procurement estimates.

### Opportunity and feasibility (capacity building)

Participants from Malawi and the Philippines indicated that domestic medical missions are now becoming more common than foreign missions. Whereas in Honduras, foreign short-term medical teams to provide primary care continue to be requested by many local government leaders, as well as community groups located in remote regions. Specialty and surgical missions are also becoming more prevalent, and current STMMs in the Philippines often consist of a variety of medical professionals who provide primary care, dental, and surgical care during the one STMM.

Coordination and continuity planning remain critical concerns for any medical mission [41]. Government officials in the Philippines urge foreign STMMs to follow “*adopt a hospital*” and “*adopt a community*” strategies to promote continuity and capacity building. Additionally, there is interest in how medical missions can contribute to local continuing professional development programs. Similarly, a government official in Honduras requested that medical missions focus on strengthening the overall sustainability of host communities’ medical systems and engage in reciprocal capacity building.

Participants in all the stakeholder groups and countries routinely identified that specialist medical and surgical services are the priority needs if health professionals arrive to volunteer in direct clinical care. In Honduras, one participant (from a university teaching hospital) speculated that the institution’s *“international affairs department could collaborate with STMM personnel on training and development.”*

### Guideline recommendations

A Malawi participant from the Ministry of Health requested *“a shorter checklist”* that would accompany the full 24-page guideline. All participants interviewed across the three countries found the current STMM guideline document onerous and difficult to use. The authors have acknowledged and responded to this feedback by creating sending and host organization checklists (Additional file 3).

There was overall acknowledgement that additional resources and tools were needed to support medical mission trips for example: health professional/volunteer code of conduct, health professional/volunteer consent and waivers, pre departure survey for host country and STMM to complete together, patient information sheet, consent, and referral form for foreign health professionals to be able to refer back into the local health system, and sample photo release consent form.

## Discussion

This study describes the perspectives of host country stakeholders regarding guidelines for STMMs. Our quality improvement project was underpinned by decolonization theory, aiming to reveal and dismantle colonial power in all its forms.

Two previous studies briefly describe stakeholders’ opinions on STMMs. Lasker et al. [5] led an analysis of the existing 27 guidelines for STMMs at the time, finding that few were written with stakeholders from host countries. The second study interviewed 450 STMM organizers and hosts and found that: volunteers on a STMM are unprepared to deal with the culture or language of the country in which they will volunteer [8]. Volunteers from high-income countries prioritize providing clinical care during the STMM, but local hosts ideally want volunteers to assist with capacity building [8]. There is also infrequent collaboration between hosts and the sending organization to improve STMMs, as shown by Lasker et al. [5], who identified only four STMM-related articles that were co-authored by medical professionals from host countries. To the best of the authors’ knowledge, this study is the first to focus on the process, rather than the results, of guideline implementation and represents an important aspect of evidence-informed practice.

One of the major strengths of this study was the total number of participants interviewed and the depth of the interviews from the three different countries. Information from these interviews was used to inform best practice guidelines development for STMMs. Naturally, these guidelines will need to be locally adapted and specific details on medical and health professional licensing made specific. Local and foreign partners will need to work together to ensure the best practice guideline is implemented well. The experiences of the participating stakeholders in this study illustrate that guidance for teams providing short-term medical aid is necessary to ensure no harm and to ultimately support and strengthen the local health care system.

It is recommended that managers of STMM teams work in conjunction with managers with the host partners to complete a needs assessment before arriving. This practice, as well as working alongside local healthcare staff, will promote collaboration throughout the entire mission and ensure improved quality of care for patients including care that is integrated into long-term systems which can support patient needs when a STMM is gone. Close collaboration during direct clinical care in a STMM also supports skill development for all health professionals involved. STMM health professionals must adhere to local licensing and legal requirements. Over time, these changes will serve to strengthen the links and trust between medical missions and the local health systems of the host countries, which will improve the way that medical missions are implemented overall. This importance of trust, and how to initiate, nurture and maintain it within these partnerships, is interwoven within the shared roles and responsibilities of the PQMD STMM guidelines themselves. One recent study pointed out “that trust is a pillar on which partnership is built upon and sustained” [43]. Furthermore, this study revealed that stakeholder participants believe that building capacity and improving access to medical supplies should also be among the primary objectives of STMMs. According to our interviewees, many STMM teams are currently focused on providing clinical care. Continuity should be a critical concern for all STMMs, so rather than providing a short-term solution or undermining the local health system, teams should direct their attention to improving the overall quality, quantity and sustainability of local healthcare and health/pharmaceutical supplies. Steps should be taken to move organizations from one-time STMMs to regular ongoing program support to facilitating sustainable in-country health systems. Some country governments are following this ideal by encouraging foreign medical missions to “adopt” a hospital or community.

As stated above, medical mission teams may also play a key role in supporting medical product needs; for pharmaceuticals, however, the differences in local formularies and overall familiarity with certain medicines must be taken into consideration.

Overall, the updated STMM practice guidelines categorizations received positive feedback from the majority of stakeholder participants. This guidance now provides more structured coordination of the entire medical mission process and has the potential to considerably improve the way that STMMs are carried out. They have also proven to be a useful tool for the actual implementation of STMMs. Additionally, there is a need to identify the minimum requirements for culturally accepted best practices, in accordance with the local culture in which a given STMM will operate [44].

As this study disclosed, there are countries whose systems are already robust, and the visiting STMM teams should respect existing regulations in place in those countries. Guidance regarding STMMs will need to be updated due to changes in technology, politics, travel, COVID-19, supply chains, and human resources.

### Study limitations

This study has some limitations. First, purposive sampling could be seen as a limitation. According to Patton (2002), this kind of sampling permits the researcher to select key individuals who have a great deal of knowledge about the phenomena of interest, which aids in the acquisition of information-rich data [45]. Purposive sampling supported timing and feasibility needs during in-country site visits by the research team. Next, the sample size varied by country, with Honduras possessing 62% of the 118 participant interviews where contacts, and networks were strongest. Malawi receives less STMMs therefore reached saturation of data earlier, as the experiences were smaller and more similar there versus Honduras where STMMs are common, therefore possibly more diverse views and therefore required greater numbers of interviews to reach saturation. Lastly, patients were excluded from this study given the length of the guidelines document and logistics of sharing by email pre interview. Also, the different local languages by country and the availability of the guidelines document available only in English and Spanish are limitations.

## Conclusion

The onus of implementing STMM best practices should be on the sending organizations rather than the hosting organizations or countries. Medical missions should support universal healthcare and the local system, not disparage or undermine either. This can be done only when there are strong links established between the medical mission team and the local health system.

A wide variety of problems exist with STMMs, including the perpetuation of colonization. The field of medicine has a long and troubling history in formerly colonized countries, but many healthcare providers and medical faculty remain unaware of this history. As a result, STMMs sustain colonization, as the balance of STMM benefits continue to favour the sending healthcare practitioners and trainees relative to the underrepresented host partner’s organization, communities, and existing programs [30]. Overall dissemination strategies from the sending countries and their respective organizations should bear the professional and ethical responsibility of improving upon and implementing changes in existing STMM models.

The PQMD-HSS/MM guidelines document is onerous. Their review and implementation will require a great deal of time, and some participants believe that putting the guidelines into practice will increase costs to medical teams and deter STMM support. Checklists, as suggested by interviewees, were created for the host and sending countries could further be tailored locally and simplified. If STMM guidelines cannot be achieved, then it is recommended that teams look toward more formally developed partnership models such as those described by the World Health Organization through formalized, collaborative twinning arrangements as an effective way to improve standard of living and achieve sustainable, long-term improvements in global health systems [46].

## Supplementary Information

**Additional file 1.** List of working group members and organizations.

**Additional file 2.** PQMD-HSS Medical Mission Guidelines.

**Additional file 3.** PQMD-HSS Host and Sending Country Checklists.

## Data Availability

The datasets used and/or analyzed, and the participant questionnaires are available from the corresponding author on reasonable request.

## Abbreviations

GDP: Gross Domestic Product
HIC: High Income Country
LMIC: Low- and Middle-Income Country
NGO: Non-Governmental Organization
PQMD: Partnership for Quality Medical Donations
PQMD: HSS/MM Partnership for Quality Medical Donations Health System Strengthening/Medical Mission
STMM: Short-Term Medical Mission
