# Supplementary material for: Guidelines for short-term medical missions: perspectives from host countries
Source: Global Health. 2022 Feb 19;18:19. doi: 10.1186/s12992-022-00815-7 (PMC8857875; doi:10.1186/s12992-022-00815-7)
Supplement: Supplementary file 1 — Additional file 1. List of working group members and organizations. [file 12992_2022_815_MOESM1_ESM.pdf]

Standards for Medical Mission Partnerships and Practices
(Sending and Host Organizations)

PQMD
Partnership for Quality Medical Donations

americares

Healthcare System Strengthening/Medical Mission Initiative

October 2019

Guidelines revised September 2018

TABLE OF CONTENTS

Sections	Page number
- Introduction	3
- Overview	4
- PQMD HSS/MM Standards	
o Assessment	5
o Partnership/Alliance	6
o Governance	7
o Code of Conduct	9
o Preparation	10
o Implementation	12
o Training and Capacity Building	13
o Sustainability	14
o Monitoring and Evaluation	15
- References	17
- Appendix A List of Definitions	19

With support from:

Dr. Julie Varughese, Americares; Dr. Patti Tracey, Trent University; Elizabeth Ashbourne, Partnership for Quality Medical Donations

Dr. Timothy Amukele, Pathologists Overseas & Johns Hopkins University; Veronica Arroyave, Baxter Foundation; Darnelle Bernier, Catholic Medical Mission Board; Doug Fountain, Christian Connections for International Health; Judy Hastert, Heart to Heart International; Samuel Ingram, Medtronic; Kim Keller, Johnson & Johnson; Carla Orner, Heart to Heart International; Dr. Anne Peterson, Americares; Juliemarie Vander Burg, Partnership for Quality Medical Donations; David Obando Venegas, Trent University; Randy Weiss, Americares; Dr. Philip Wendschuh, North Ohio Heart Ohio Medical Group; Julie Winn, Americares

Myron Aldrink, Partnership for Quality Medical Donations; Dr. Sarah Brown, Pathologists Overseas & Washington University; Wade Jones, Medtronic; Claudia Sighomnou, HPIC Canada

INTRODUCTION

Medical Mission – Current Practice

The literature reveals that Short-Term Medical Mission contributions are valued yet fragmented in their current state with largely unknown outcomes or well documented practices. On the positive side, medical missions provide valuable medical services to people who might not have access to healthcare otherwise. This is especially prevalent in rural and remote regions where poverty may be extreme. Medical missions also provide benefit to the volunteers themselves by gaining personal satisfaction from participating in humanitarian efforts and an opportunity to reconnect to why they chose to pursue a health focused profession. Medical missions can also support student experiences as they gain exposure in the field of international medicine through opportunities as part of their education.

Challenges occur however when doctors and other health care professionals from high-income countries (HIC) demonstrate a lack of awareness about the realities of healthcare in low middle – income countries (LMICs) or show a lack of respect for local health workers. Often because of the short-term nature of MMs, visiting health care providers often have little time to dedicate to understanding local health needs and culture. Furthermore, insufficient attention is given to imminent local issues, adequate follow-up and ongoing care – often not knowing the local health system well enough to refer patients for ongoing care. Short-term medical missions are also often criticized for not being the best use of financial resources.[2] An average medical mission cost is $USD 41,359 -65,000 for a team of 20 people (Tracey, 2015).

The number of medical professionals participating on medical missions is large and increasing. It is estimated that annually, over 16% of US doctors go on medical missions with medical mission costs and related expenses totaling over $US 3.7 billion (Caldron, 2016).

PQMD Medical Mission Standards - Purpose

PQMD recognizes the dilemma regarding short-term medical missions and understands that medical missions can be beneficial and provide appropriate and quality health services. PQMD further believes that medical missions should help support and strengthen local healthcare systems. Therefore, in May 2016, PQMD announced the 3-year initiative called the PQMD Healthcare System Strengthening/Medical Mission Initiative (PQMD HSS/MM). This initiative reviewed the complex nature of medical missions and the relationship between different stakeholders. It noted that many of the problems seem to pertain to lack of alignment between:

- Sending Organizations (NGOs, churches/religious organizations and universities)
- Host Organizations (INGOs, In-country churches/religious organizations and universities)

Therefore, PQMD and supporting member stakeholders have developed a set of standards that seek to better align the purpose and practices of sending and host organizations. The standards are intended to help create common understanding of directives of sending and host organizations and to help form better partnerships. They provide high-level principles for improving the quality of medical mission practices including the encouragement to provide training and capacity building. These standards are not intended to be draconian and judgmental in nature but instead to encourage improvement in medical mission efforts over time.

OVERVIEW OF STANDARDS

The PQMD standards are based on the following premises:

Support for the World Health Organization
The PQMD medical mission standards are designed to support World Health Organization (WHO) directives.

Health Development Focus (not Disaster Relief)
The PQMD standards pertain to long-term health development - not disaster relief efforts. The WHO has established standards for foreign medical teams for disaster relief (*2013 Classification and Minimum Standards for Foreign Medical Teams in Sudden Onset Disasters*). However, the WHO does not currently have standards regarding medical missions for long-term health development.

Healthcare – Area of Emphasis
The PQMD Initiative is focused on primary care however, the standards are designed to be general principles applicable across medical disciplines.

Medical Mission trip – Time Durations
Most medical mission trips are short term (4 weeks or less). However, the PQMD standards are also applicable to medium-term mission (5 weeks to 6 months) and long-term missions (7 months to 2 years). (Martiniuk et al, 2012).

Local Health Systems - Scope
The PQMD standards cover medical missions supporting the entire healthcare system (Primary, Secondary and Tertiary facilities). Note: Some medical missions are conducted in churches, schools and rural community facilities, these locations pose challenges as they typically are not connected to the healthcare system.

Medical Mission efforts- Increasing Commitment
The standards also encourage increasing the level of commitment towards sustainable local health programs. The standards include steps to move organizations from one-time medical mission trips → to regular ongoing program support → to developing sustainable in-county health operations. These different levels of commitment are described below.

- One-time medical mission trips or infrequent trips. Driven by the desire to conduct trips to serve an appropriate local need when volunteers can be organized to serve. These one-time trips are typically medical service oriented.
- Regular ongoing trips and program support. Driven by the desire to provide regular, dependable, ongoing support for local health programs. These trips are closely coordinated with the host to provide support as needed. These ongoing trips have a greater emphasis on providing training and capacity building.
- Permanent In-country health programs. These ongoing local efforts often have local boards. However, these programs are augmented by occasional medical mission trips Driven by a commitment to create sustainable locally run programs (without dependency) these permanent programs are typically well integrated within the local healthcare system

PQMD HSS/MM STANDARDS

The PQMD HSS/Initiative has created a set of standards designed to better align sending organizations and host organizations. The **9 PQMD standards** outline the general principles to help create partnership and mutual respect. These standards also focus on improving the process and practice of medical missions and programs including training and capacity building efforts.

1) ASSESSMENT

The essential first step is to conduct a general assessment to determine if a medical mission effort is appropriate and viable. There are different types of assessments, including: population assessments, health assessments and needs assessments. Pre-trip assessments also include a costs and benefit analysis.

While assessments are customized to the specific programs and type of medical intervention, the general need to obtain upfront information applies to all medical disciplines and groups (NGOs/INGOs, small churches/religious organization, Universities, individual volunteers etc.). The key to success is for the host organizations to be an active partner and genuinely involved in these assessments.

Sending Organization

- Work with host to assess population profile
- Clarify and understand health situation/culture
- Develop an awareness of people living with disabilities in the communities where the MM is taking place (are services accessible?)
- Work with host on health/wellness assessment
- Ascertain resources available to build capacity
- Identify where local assets are weak or missing
- Assessment of the capabilities of in-country heath facilities (both host organization and local health system)
- Work with host on program needs assessment-consider pre-trip assessment to check:
    - Specific health program needs
    - Facility needs (capabilities/management)
    - Staff capability (training to local needs)
    - Equipment (appropriate/biomed support)
    - Products (consistent with local supply, availability; for example: what is prescribed can be procured /refilled locally)
- Work with host to develop the needs for MM trip (Determine whether the medical mission assessment will be conducted virtually or in-

Host Country Organization

- Describe local population (social, economics etc.)
- Provide history/context of local health system
- Identify and prioritize health and wellness needs
- Describe local resources currently available and help determine needs (asset mapping)
- Assessment of training needs (medical, process, IT, biomedical training etc.) as they relate to current and future program goals.
- Provide current and future program goals
- Describe program and local health system needs
- Describe building, process and staff situation
- Describe staff size, experience and skill level
- Describe equipment/consumables/costs
- Describe local products/ cost /supply network
- Provide information on local programs for coordination with medical mission effort
- Explain in-county health structure
    - MoH – National Health Plan
    - Local Healthcare structure
    - Local Community health capabilities
- Describe approval process of government

person)
- Work with host to understand national health requirements, local health system and local community health efforts
- Assessment of available personnel and their skill levels for the planned medical mission
- Ensure that each volunteer position helps with the overall mission goals and objectives.
- Estimate the costs and benefits of MM effort
  - o Health services costs
  - o Training costs
  - o Capacity building costs
  - o Benefit to sending organization reputation
  - o Benefit to individual volunteer (personal growth)
  - o Cost/benefit assessment of the entire MM trip
- Assess costs of other resources required for example:
  - o Logistics/Accommodations/travel
  - o Language translators
  - o Volunteer orientation/prep (e.g. vaccination)
  - o Contingencies e.g. In country Emergency
  - o Organization, protection and liability

for volunteers -e.g. registration/medical licenses product/equipment (e.g. local consumables) immigration requirements/custom fees etc.
- Assessment of the medical mission and the potential impact of the program on staff and local community.
- Assess benefit of MM to host organization
  - o Benefit of medical service to local program
  - o Benefit of skill training to local staff
  - o Benefit of resources/ process training
- Protect reputation of host and local health system
- Note: the planned medical mission must enhance not demoralize or diminish confidence in host organization or local health system.
- Assess overall cost and local resources required to determine if MM trip is the best use of the limited resources of host organizations and local health system

2) PARTNERSHIP/ALLIANCE

If the assessments indicate a medical mission would be beneficial, the next step is to develop a formal partnership where the parties agree on the relationship and activities. This doesn't have to be a long complex legal document, but it is very important that expectations, responsibilities and cost be clearly understood and agreed upon by all involved through the joint development of a memorandum of understanding (MOU). To be successful, medical mission partnerships must be aligned with the overall goals of all parties.

Sending Organization
- Present sending organization mission/purpose
- Discuss general interest in partnership (short and long-term interest)
- Discuss planned medical mission trip(s)
- Obtain general agreement on scope of program
- Engage in collaborative goal setting with host

Host Country Organization
- Present host organization mission/purpose
- Discuss interest and host requirements
- Discuss past history with other partners
- Discuss local expectations of MM trip
- General agreement on program - local impact
- Provide program expectations
  - o Specific benefits

- Define expected learning/teaching outcomes
- Determine expected patient outcomes (treatment and/or prevention)

- Confirm duration of the mission with host
- Define local program resources required (facilities, patient access, staff support etc.)
- Do not pay for services/staff that is beyond the local norms.
- Recognize cultural differences in conversations
- Selection of MM participants (select participants based on certain criteria such as, understanding of the language, level of awareness about the destination country/community, cultural familiarity, required specific skills, past experience, etc.)
- Identify lead of MM team and HQ authority
- Define financial/resource allocation
- Define reporting and measurements
- Agree on terms for MM partnership review
- Consider further partnership and longer-term partnership agreement
- Define the level of information (confidential and non-confidential) that will be shared from sending to host (financial assessment/impact, funding prospects, relevant personal team member information, etc.)

  - Specific benefits from training
  - Patient care and health benefit
- Confirm duration of the mission and plans to coordinate with local program schedule.
- Define resources to be provided, received and used (services, training, product/equipment etc.)
- Seek cultural understanding /seek respect
- Identify host and local individuals that would best fit with medical mission team
- Identify lead person for medical mission interface (support for sending organization)
- Conduct government pre-approval efforts (in conjunction with sending organization)
- Agree on in-country use of finances/ resources
- Plan for obtaining information and reporting
- Agree on local review process for MM effort
- If MM is positive, seek further partnership commitments (with goal of self-sustainment)
- Define the level of information that will be shared with sending organization – explain local and cultural sensitivities.

3) <u>**GOVERNANCE**</u>

Prior to any joint medical mission efforts, both organizations should provide each other with proof of due diligence and governance compliance. Due diligence proves that the organization is properly managed and can stand up to scrutiny. Governance pertains to compliance with national laws (both sending and receiving) and local regulations. In addition, the organizations should show adherence to global principles and ethical practices.

The actual documentation regarding due diligence may differ based on country and organization category. For example, organizations may differ in structure (NGOs, churches/religious organizations, universities informal volunteer groups); however, the same general principles of accountability and transparency apply to all organizations (both sending and host).

Laws and regulations vary by country. This document presents North American examples however, the global equivalent would apply for all participating HICs. It should be noted that in some countries the government is under-resourced and may have difficulty with enforcement and support. However medical mission volunteers and programs should comply

with the laws and regulations of the host country. Also, sending countries may have different laws however, in many instances the laws are similar. Overall the spirit of due diligence and good governance is a major element in the general principles of medical missions.

Sending Organization

Due Diligence

Provide host with due diligence documents (legal entity, financials, tax status)
- Bylaws/List of Directors
- 501 C 3 tax status (US)
- Canadian Revenue Agency (Canada)
- Audited Financial/ 990 /GAAP (US)
- T3010/IDE(Canada)

Legal compliance

Discuss with host legal requirements, including:
- Foreign Corrupt Practices Act (US)
- Corruption of Foreign Public Office ACT(Canada)
- Office of Foreign Assets Control (US)
- Minister of Foreign Affair (Canada)
- Protected Health Information HIPPA (Patient Privacy (US)
- Medical mission teams must know laws of the host country and be in total compliance (not just compliance with laws of sending country)

Confirmed ethical compliance

Discuss with host basic expectations regarding:
- Human Rights Principles (UN)
- Patient Safety (WHO)
- Healthcare Compliance (US Office of Inspector General)
- SPHERE – Quality Humanitarian Response (Red Cross/Red Crescent and NGOs)
- PQMD – Partnership for Quality Medical Donations – Product donation standards
- THET – Tropical Health Education Trust (UK Organization – strengthening healthcare)

Program Health Role Compliance

In addition, sending and host organizations must agree on program health role and practices
- Define and establish scope of practice for each category of health practitioners
- Define and establish scope of practice for non-health participants

Host Country Organization

Due Diligence
- Agreement on governance documents
- Provide sending organization with documents of host organization (viability of entity, financials
- Confirm host organization is in good standing with in-country government and local authorities

Legal compliance
- Confirm an understanding of the requirements of sending organization regarding corruption and health laws and that the host organization is in compliance
- Provide sending organization with basic local health program practices (e.g. patient data sharing, cultural norms patient privacy expectations)
- Clearly communicate local laws and also informal practices regarding these laws

Confirmed ethical compliance
- Explain local cultural and ethical practices/concerns
- Communicate a basic understanding of the expected ethical standards of sending organization and agreement to comply
- Confirm that the sending organization also understands the ethical expectation of local country and host organization.
- Describe local product supply network with economic and social considerations
- Convey ethical sensitivity and respect for local healthcare practitioner roles

Program Health Role Compliance
- Define and establish scope of practice for MM volunteers both health practitioners and non-health participants
- Confirm the scope of practices is in compliance with in-country health laws and regulations
- Confirm student /trainee involvement is in

- Define and establish scope of practice for each category of health students/residents/trainee
- Define required level of supervision needed for student/trainee/resident participants

compliance with local health laws

4) **CODE of CONDUCT**
Many problems regarding medical missions pertain to attitudinal issues. It is essential to show humility and respect for all involved. Cultural sensitivity plays an important part in the success of medical missions. Therefore, the following components should be considered:

Sending Organization

- Establish general organizational Code of Conduct policy for MM volunteers based on WHO directives:
  - Patient Safety
  - Healthcare System Strengthening
  - Training/Capacity Building
  - Patient Monitoring /Health Impact
  - Child Protection
  - Gender
- Define anti-discrimination policies (race, nationality, religion, creed, political standpoint)
- Consider factors which might affect rapport with any team member/patient or local community member)
- Establish rules of respect for host organization programs and culture (appearance and work attire)
- Consider sexual exploitation and abuse in Humanitarian Settings (IASC Task Force, 2016).
  - Avoid paternalistic attitude
  - Comply with local health practice
  - Support local health workers
- Practicing within the scope of volunteer's licensure and preparation and not acting beyond competence (this includes students)
- Promote patient autonomy by obtaining informed individual consent from patients. (collaboration with host may be necessary to achieve this)
- Be sensitive to potential economic impacts
  - Don't hire away local health workers
  - Avoid negative impact on local

Host Country Organization

- Define Code of Conduct for local program, especially workers involved with the mm effort
- Describe expected behavior for medical mission visitors (Humility, respect, cultural sensitivity)
- Establish group norms and awareness around safe conduct
- Create a forum for candid opinions/concerns such as safe conduct
- Communicate local social norms and religious considerations
- Seek to provide good experience for mm guests share pride in local culture
- Provide information on local practices (rationale)
- Explain local health structure/processes
- Make formal introductions to key local health
- Establish patient selection process around patient recruitment and respect
- Describe local health economics/implications
- Explain staffing and health system needs
- Discuss local financial considerations of host organization and healthcare systems Discuss proper use of assets
- Convey support for local healthcare system
- Explain the major political and social situation and define sensitive topics that guest should not engage or make comment.
- Provide information on local environmental practices.

healthcare system (i.e. providing “free” medical services)
    - Avoid causing diminishing confidence in local health system
- Be sensitive to level of interference with local issues that are not medically related or medical mission related – e.g. sensitive to local politics by outside organizations
- Consider environmental impact of medical missions (litter, disposal of medicines and other hazardous items, etc.)
- Obey all ethical and global governance requirements
- Human Rights
    - Patient monitoring /Health impact
    - National health rules
        - local medical license
        - register as MM practitioner
        - government directives/policies
- Code of conduct on dealing with government officials/military, in times of war/emergencies
- Code of conduct on giving bribes, kickbacks or other improper payments
- Code of conduct for accepting and offering gifts (feeling the urge to help with money and pay your way out of process, giveaways such as toys or footwear, clothing etc.).
- Code of conduct with regards to safety and security. Define course of action in case of adverse event reported
- Consider course of action if medical lifesaving practice are in opposition with strongly enrooted cultural norms
- Code of conduct in emergency situation.
- Code of conduct with regards to confidential information and privacy, conflict of interest or social media guidelines
- Images, logos and other intellectual property (taking and using photographic images)
- Other Considerations
    - Conflict of interest
    - Sexual exploitation and abuse

- Explain local social and humanitarian concerns
- Explain local patient care and recording
- Explain local health reporting processes
- Explain local government policies and requirements
- Assist mm team with obtaining registrations
- Assist sending organizations to deal with any local government crisis
- Explanation of local corruption and advice to maintain ethical practices
- Clear rules should be established in both sending and receiving countries/communities on how gifts should be handled
- Assist sending organization in the event of local safety concerns
- Assist sending organization with any local lifesaving or emergency situation
- Coordinate with sending organization regarding local communication practices
- Have open communication with sending organizations regarding any inappropriate social activities

5) <u>PREPARATION</u>

Advanced planning and preparation is very important for medical missions. It is especially important to develop plans in conjunction with the host organization

Sending Organization

- Continued communications with host on medical mission plans and objectives including site selection and role of all partners
- Create a Memorandum of Understanding (MOU) with host partner(s)
- Define selection criteria for volunteers
  - Credentials
  - Technical Skills
  - Medical mission experience
  - Pre-departure training
  - Personality fit
  - Cultural attitudes/sensitivity
- Confirm program resources required
  - Health practitioners
  - Facility/Location
  - Product/Equipment
  - Technology
  - Respectful, educational photo taking only (with patient consent)
- Work with the host community to determine the issues around equity, human rights, gender and other social determinants of health.
- Coordinate logistics from departure to return - with emergency contingencies.
- Plan participants safety
  - Vaccination and immunization
  - Security and safety measures around local travels
  - Food safety (dietary, allergies)
  - Locate consulate/embassies
- Learn destination's national formulary and become familiar with local medical products and foods available locally

Host County Organization

- Discuss in-country program plan (clarify objectives and expectations)
- Mutually agree on the timeline of the project and finalize local program itinerary
- Determine preferred mode of communication, work with contact person and identify resource
  for MM team prior to their arrival
- Establish rules and local expectations
- Prepare country/community immersion and orientation programs for MM program participants (both sending and local host) or team members - possibly matching key people for better familiarization with local skills
- Define nature of work and anticipated skills tools needed
- Describe the capabilities of the facilities/location
- Confirm product needs and local appropriateness
- Provide information on local tech capabilities
- Describe local social/political/economic situation
- Plan for all necessary legal permissions in host country (legal authorization to import donated medicine, medical mission practice by foreign health professions)
- Prepare adequate accommodations/local logistics for teams
- Inform team in advance of current health and safety conditions plus expected weather/floods
- Confirm the number of participants that can be hosted, given food, and accommodation
- Understand in advance the composition of incoming team
- Provide information on local staff and perceptions
- Plan ahead for all local resources required to the team (facilities, skilled health practitioners, technology, products, equipment, interpreters)
- Determine local health system interface
- Prepare for announcement of the arrival of type of team for the targeted community (especially community leaders), and make sure of cooperation, consent and commitment

6) IMPLEMENTATION

The implementation of the medical mission will vary by the specific organization/program. This includes the execution of the current medical mission effort, but should also include planning ahead for possible future medical/health programs and support for local healthcare system strengthening

Sending Organization

- Work with the host to implement the trip /program as planned
- If changes required, work with host on changes
- Have regular communication with host during implementation to gauge progress
- Utilize local health system
  - Don't run parallel efforts
  - Avoid disrupting regular flow of service delivery
  - Prescribe local medicines from trusted sources
- Prepare site upon arrival
  - Meet and get to know the local team
  - Determine the number of patients to see per day
  - Optimize patient care (take account of language barriers)
- Implement medical procedures that conform to accepted local standards if not, international standards.
- Work to address local issues, such as:
  - Laboratory without Biomed equipment, techs and repair service
  - Surgical without functional facilities /trained staff/local supplies
  - Primary Care without health system interface
  - Cost effective local pharmaceuticals and med supplies for long-term care of patients
- Provide/dispose of product and equipment in accordance with PQMD Guidelines and all other standards and directives
- Conduct a final project review with host at the end of the MM trip
- Discuss long-term continuity

Host County Organization

- Implement the MM effort per plan and work within local, existing programs (when possible)
- Provide support and advice if the MM plan changes especially when local solution needed.
- Provide on-going communication and local feedback on MM activities during implementation
- Provide local resources coordinated to best interface with existing healthcare system
- Work with sending organization to coordinate programs which "builds-off" local facility capacities
- Implement the patient process and prepare patient files (patient recruitment/prep)
- Establish a referral system to follow up on patients seen during the medical mission
- Organize hand-off sessions
  - Patient needing follow up care
  - Collect patient information sheets
  - Collect comments and feedback
- Establish inventory management procedure to ensure appropriate use of medicines (labeling, expiry dating, etc.)
- Provide information on local capabilities/ supplies
- Establish joint reporting on patients for follow-up purposes (especially for chronic diseases), and carefully file patient information and statistics
- For monitoring and referral purposes, notify the local health center of the efforts of the medical mission in their areas of responsibility
- Support cleanliness of community and local facilities and support product network and disposal needs

- o Multiple trips to same location
- o If possible, and appropriate, work toward establishing in-county program

- Debrief on medical mission program activities on a regular basis
- Reviews and critiques going "both-ways" provide perspective from host and health system
- Encourage long-term continuity to develop local health capabilities (not dependency)

7) TRAINING /CAPACITY BUILDING

Medical missions should be viewed as "temporary" with the ultimate goal to develop local health system to where it can take care of itself. Medical missions are "gap fillers" however, they can help build towards the future by providing transfer of knowledge (training and capacity building) – with the ultimate goal of having sustainable local programs and a viable a strong healthcare system.

Sending Organization

- When possible, provide training in addition to MM clinical services including illness prevention education (By med students only when appropriate)
- Seek to coordinate health training with local workforce and officials
- Try to develop training in conjunction with local educational institutions (College/medical organization certification)
- Discuss expectations and responsibilities of both host and sending institutions and agree on terms of training program implementation
- Develop structured program plans that include bidirectional training and local capacity building interaction
- Prepare a detailed outline on how the training will be conducted such as clinic/hospital shadowing, specific training sessions, simulations, etc.
- Promote transparency regarding the motivations for using program as training opportunity and identify and address any conflicts of interest that may result from such a program
- Clarify the level of training and experience for the host institution so that appropriate activities are assigned, and patient care and community well-being is not compromised
- Establish effective supervision and mentorship of trainees with the host and

Host County Organization

- When possible, seek opportunities to obtain training for local health practitioners and staff
- Make introductions to local health officials medical associations, and government officials
- Make introductions to in-country education system government agencies and medical associations
- Include local health systems and government agencies in discussions regarding training efforts and long-term coordination
- Proactively provide local knowledge and health experience to sending organizations
- Achieve local awareness, understanding and buy-in for any training effort
- Seek up-front orientation of planned training efforts and discuss impact of these efforts on the community and local health system
- Emphasize the importance of training methods appropriate for host country (cultural sensitivity)
- Involve all government agencies that approve and certify health training and education
- Include key local officials in training

including the selection of appropriate mentors and supervisors and facilitate regular communication among them.
- Assess the cost and benefits to host institutions, local trainees, patients, communities, and sponsoring institutions to assure equity.
- Establish methods to solicit feedback from the trainees both during and on completion of the training program. Ideally, follow up after 3-6 months to see if the training has had any impact.
- Determine ways to measure transfer of knowledge and application to local care
- Determine the local assets that are available to assist with competency development and capacity building
- Provide initial resources to help develop local capabilities (i.e. IT, medical equipment and technology)
- Develop capacity in conjunction with local health system and medical associations, government officials and WHO plan
  - Leadership/governance
  - Health workforce skills
  - Health facility and process capabilities
  - Medical equipment (biomed support)
  - Medical products and process
  - Financing capabilities
- If requested, provide some on-going support (with time limits to avoid dependency)

development both health professionals and community leaders
- Seek local involvement in all levels of the training aspects.
- Proactively provide local knowledge and health experience to sending organization and training efforts
- Work with sending organizations to develop appropriate local skills attainment metrics (in conjunction with government agencies)
- Determine the local assets that are available to support competency development and capacity building
- Seek engagement in local capacity development planning and implementation bringing in local health officials and individuals with support skills
- Seek long-term partnership with health officials
- Seek to obtain local funds/ partial fund training and capacity building activities (a balanced partnership)

8) SUSTAINABILITY

Training and capacity building can help improve local health efforts, but to be sustainable requires locally managed and properly funded health efforts. Long-term collaborative relationships can help move towards sustainability.

Sending Organization

- Work with host to establish long-term collaborative relationships
- Focus on prevention and development efforts - not “treat and leave”
- Select local partner organizations that have long-term presence locally and can provide follow-up and ongoing care
- Establish short-, medium-, and long-term measures of success (number of patients

Host County Organization

- Work with in-country authorities to support these long-term efforts
- Work with the community and health system officials to establish local prevention efforts
- Advocate to develop local capacity to transform medical mission experiences into sustainable output across the health system (primary, secondary and tertiary

seen, prescriptions given, incidence, prevalence or control of a disease, to measure and improvement long term access and support and strengthen the local healthcare system
- During the transition period (after the MM trip) carry out a responsibility transfer process where medical and patient information will be passed on to local hosts
- If several medical missions planned, consider recurrent medical missions to the same location
- Seek to develop multi-partner multi-sector collaborative efforts
- Foster independence by providing training and building local capacity
- Define revenue allocation between all partners focusing on developing capacity development efforts
- Work with host to expand mm health commitment – moving up One-time medical mission trips
  - On-going trips to same location
  - In-country based programs with occasional HIC teams

and local communities)
- Seek to bring in partners for long-term collaboration (local and international partners)
- Work within local political environment to obtain support to long-term efforts
- Seek local responsibility and resources to create a more balanced partnership relationship
- Develop programs with business logic (not charity)
- Seek to establish local joint effort for long-term sustainability (e.g. merging local partners)
- Define revenue allocation between all partners
- Consider self-sustainable revenue stream to compensation practices and support pay for expatriate and national (this would limit public-private pay inequity, strengthen public sector, incentive for rural service)
- Seek continuity (rather than only 1 trip)
- Seek formal in-country programs (in conjunction with the MoH and local healthcare system)

9) MONITORING and EVALUATIONS

Finally, there must be monitoring and evaluation of medical missions and health efforts including measurement of outcomes and health impact metrics. Measuring activities are needed to gauge progress and, equally important, help identify areas to be improved for long-term sustainability. While measurement methodology will differ for individual programs, the basic measurement principles are summarized below:

Sending Organization
- Work with host to define and monitor objectives and conduct program evaluations on MM trip
- Create program health metrics to evaluate the current MM effort
- Work with host to establish criteria for overall health performance measures
- Conduct reviews of the MM program relative to in country health metrics
- Assess cost-benefit/effectiveness and financial viability of medical mission

Host County Organization
- Provide information on overall local measurement practices and procedures
- Advise on measurement practices currently used by local program
- Provide information on healthcare metrics currently used by local healthcare system
- Coordinate and support with data collections for MM program and national health efforts
- Work with the sending organization to

- Create basic metrics to help develop future MM efforts and program improvements
- Utilize standard measurements such as; input, output, and outcome/health impacts

Inputs
- o Amount of funds spent
- o Number of medical volunteers
- o Amount of product/equipment

Outputs
- o # Services provided/patients
- o # of people trained
- o # of facilities improved

Outcomes/Health Impact
- o % population covered
- o Health impact (morbidly/mortality)
- o Improvements in markers such as hypertension and blood glucose
- o Economic impact
- o Patient satisfaction
- o Patient follow up and referral

- Create ongoing measurement regarding ethical and health directives (WHO, UN etc.)
- Develop information and establish basic metrics regarding training and capacity building efforts (including baselines)
- Develop specific measurements for report to donors to document on donations (cash and in-kind) used in the program
- Also provide qualitative personal reporting in the program review
  - o Debrief – Post MM trip review
  - o Documentation of collaborative future plans and identified health care needs

provide cost and health information specific to MM
- Assist in the assessment of future programs potential with health impact and costs consideration

Inputs
- o Calculate the benefit of funds to local program
- o Assess impact of volunteers on local health effort
- o Determine appropriateness and practical use of product/equipment

Outputs
- o Impact on patients and health program
- o Impact of training on improving local health system
- o Impact on improving local capacity building

Outcomes/Health Impact
- o Use local health impact information if possible
- o Work with sending organization to obtain specific health information requested
- o Also measure local social and economic impact

- Coordinate information on local social, health and economic measurement (when possible)
- Work with local healthcare system officials to assess training and capacity building efforts
- Provide data and information specific to request of sending organization/donors.
- Assist in providing pictures and information to be used only with consent, according to sending country norms.
- Debrief sending organization on MM effort from local perspective
- Use information/data to convey appreciation with appropriate cultural sensitivities
- Work to provide information on next steps

Finally, the measurement, evaluation and reports should include narrative describing how current medical mission effort(s) will be leading towards the long-term goal of improving and creating locally sustainable health programs. This narrative should also include how the partnership of sending and host organizations can improve local health system including training and capacity building efforts. It is also important that the report include attitudinal aspect regarding mutual respect for all involved, with a special focus on the patient.

References

Bielefield & Cleveland, 2013 Faith- Based Organizations as Service Providers and Their Relationship to Government, Non-Profit and Voluntary Sector Quarterly https://doi.org/10.1177/0899764013485160

Caldron P., Impens, A., Pavlova, M., Groot, W. (2016) Economic assessment of US Physician participation in short-term medical missions Globalization and Health https://doi.org/10.1186/s12992-016-0183-7

Inter-Agency Standing Committee (IASC), 2016). Best Practice Guide Inter-Agency Community Based Complaint Mechanism **https://interagencystandingcommittee.org/accountability-affected-populations-including-protection-sexual-exploitation-and-abuse/documents-50**

Martiniuk, A et al . (2012) Voluntourism: The downside of medical missions ABC News http://www.abc.net.au/news/2012-06-12/negin-and-martiniuk---voluntourism/4064550

Tracey, P (2015). NGOs Impact on Health Care Services in Rural Honduras: Evaluating a Short-Term Medical Mission (STMM) Utilizing a Case Study Approach https://tspace.library.utoronto.ca/handle/1807/71357

WHO – Documents and Directive

The PQMD Health System Strengthening/ Medical Missions Standards were developed in accordance with the following World Health Organization Directives:

World Health Organization – Everybody's Business
Strengthening Health Systems to Improve Health Outcomes
WHO's Framework for Action 2007
http://www.who.int/healthsystems/strategy/everybodys_business.pdf

World Health Organization – Monitoring the Building Block of Health Systems:
A Handbook of Indicators and their Measurement Strategies 2010
http://www.who.int/healthinfo/systems/WHO_MBHSS_2010_full_web.pdf

World Health Organization – Human Resources for Health: Toolkit for Monitoring Health Systems Strengthening (2009):
http://www.who.int/healthinfo/statistics/toolkit_hss/EN_PDF_Toolkit_HSS_HumanResources_oct08.pdf

World Health Organization – World Alliance for Patient Safety 2005
http://www.who.int/patientsafety/en/brochure_final.pdf

World Health Organization – People-Centered Healthcare: A Policy Framework (2007)
http://www.wpro.who.int/health_services/people_at_the_centre_of_care/documents/ENG-PCIPolicyFramework.pdf

World Health Organization – Partnership for Safer Health Service Delivery: Evaluation of WHO African Partnerships for Patient Safety 2009-2014
http://www.who.int/patientsafety/implementation/apps/evaluation-report.pdf

World Health Organization – WHO Guidelines for Safe Surgery 2009
WHO Safe Surgery Checklist 2009
https://www.ncbi.nlm.nih.gov/books/NBK143243/pdf/Bookshelf_NBK143243.pdf
http://www.who.int/patientsafety/safesurgery/tools_resources/SSSL_Checklist_finalJun08.pdf

World Health Organization – The World Health Report 2008 – Primary Health Care: Now More Than Ever.
http://www.who.int/whr/2008/whr08_en.pdf

World Health Organization – Laboratory Biosafety Manual 2004 – Third Edition Primary
http://www.who.int/csr/resources/publications/biosafety/en/Biosafety7.pdf

World Health Organization – Classification and Minimum Standards for Foreign Medical Teams in Sudden Onset Disaster
http://www.who.int/hac/global_health_cluster/fmt_guidelines_september2013.pdf?ua=1
World Health Organization – Laboratory Biosafety Manual 2004 – Third Edition Primary
http://www.who.int/csr/resources/publications/biosafety/en/Biosafety7.pdf
World Health Organization- Laboratory Assessment tools 2012
http://www.who.int/ihr/publications/laboratory_tool/en/

World Health Organization-Strengthening Laboratory Management towards Accreditation
http://www.who.int/tb/laboratory/afro-slipta-checklist-guidance.pdf

Appendix A: LIST OF DEFINITIONS

The following definitions are provided to assist in the understanding of the terminology used in the PQMD Standards.

- **Audited Financials -** are financial statements which have been prepared in accordance with the financial standards of a country and have been audited by a qualified independent auditor and includes notes to state whether or not the entity is in compliance with financial requirements.

- **Form 990** (officially, the "Return of Organization Exempt from Income Tax") is the service form of United States Internal Revenue that provides the public with financial information about a nonprofit organization.

- **T3010** (officially the "Registered Charity Information Form") is the Canadian Revenue Agency form that provides the public with financial information about a nonprofit organization.

- **GAAP** (Generally accepted accounting principles) are a collection of commonly followed accounting rules and standards for financial reporting adopted by the US Security and Exchange Commission.

- **IFRS** (International Financial Reporting Standards) are the collection of standard accounting rules and Standards followed in Canada and many other nations.

- **501 (C) 3** is a name used for US tax-exempt organizations (charity organizations). The name refers to the section of the United States Internal Revenue Code (26 USC Section 501(c) which grants tax free status to charity organizations which comply with the rules therein.

- **Canadian Revenue Agency** is the government department that qualifies and regulates charity/tax exempt organizations in Canada. To be recognized a tax exempt the organizations must be registered with the Charities Directorate of the Canada Revenue Agency.

- **Biomedical Equipment Technician** is a trained individual, usually in electro-mechanic, that ensures medical equipment is well-maintained, properly configured and is functioning safely.

- **Bylaws** is a set of rules used by most charity organizations to provide governance, structure and establishes procedures to regulate itself. Bylaws derive their authority by being tied to some higher authority generally a legislature or some other government body.

- **Capacity Building (healthcare)** is the process where efforts are made to strengthen the skills, competencies and abilities of people, facilities and organizations in developing societies so they can overcome causes of suffering. The capacity building process begins with understanding the obstacles that inhibit entities from realizing their development goals. Then enhancing the abilities and physical facilities that will allow them to achieve measurable and sustainable results.

- **Code of Conduct** is a set of rules outlining the social norms and rules and responsibilities of an organization. This includes principles, values, standards, or rules of behavior that guide the decisions and procedures of an organization in a way that 1) contributes to the welfare of its key stakeholders, and 2) respects the rights of all people affected by its operations.

- **Cultural Sensitivity** Cultural sensitivity is being aware that cultural differences exist between people without assigning them a value – positive or negative, better or worse, right or wrong. It simply means that you are aware that people are not all the same and that you recognize that your culture is no better than any other culture. A challenge for

members of dominant cultures.

- **Due Diligence** is a systematic investigation of an organization prior to entering a relationship. Due diligence can be a legal obligation, but the term more commonly applies to voluntary investigations. Due diligence typically leads to more informed decisions by ensuring that the information collected in done in a complete quality organized manner.

- **Foreign Medical Teams:** groups of health professionals (doctors, nurses, paramedics etc.) that treat patients affected by an emergency or disaster. The WHO established the standards for medical mission teams in the *2013 Classification and Minimal Standards for Foreign Medical Team document.* These standards also emphasize that teams be properly trained and self-sufficient so as not to burden the national system.

- **Ethical Standards refers to commonly accepted principles that encourage the values of trust, fairness and benevolence. Ethical standards are broad statements, open to interpretation and not easily enforceable. However, the ethical directives by organizations such as the United Nations /World Health Organization (WHO) are generally accepted worldwide. It is every health care providers obligation to follow and adhere to the specific regulations and professional practice standards set by their respective college or governing body by country/province/state.**

- **Faith-based organizations** are charitable organizations whose values are based on religious or spiritual beliefs and have a mission based on the social and cultural values of their particular faith. Many faith-based organizations have health ministries and are active on an international scale including medical missions (Bielefield & Cleveland, 2013).

- **Foreign Corrupt Practices Act (FCPA)** is a US federal law that pertains to ethical requirements for any organization or individual involved with foreign activities. The FCPA has two provisions, the first addresses accounting transparency requirements; the second concerns bribery of foreign officials. **Corruption of Foreign Public Officials** is the Canadian Law which pertain to ethical dealings with foreign officials.

- **Health Information:** is the data and process that provide reliable and timely knowledge for management decisions regarding health services and the management of the healthcare systems. Health information also provides data and knowledge to assess health determinants and health status.

- **Health Services:** include primary, secondary, and tertiary care, ranging from promotion and protection of health to hospital, rehabilitation and palliative care services (Stamler, Yui & Dosani, 2015).

- **Healthcare Systems** is the organization of people, institutions, and resources that deliver health care services to meet the health needs of the population. Each nation designs and develops their individual health systems in accordance with their needs and resources.

- **Healthcare Training** is any activity focused on teaching medical professionals and support staff on methods that can be used to analyze and improve health quality. This also include clinicians, community health workers and health managers. Healthcare

training is designed to improve the skills, competencies, clinical experience, required to meet the health needs of the populations they serve.

- **Health Workforce:** are the medical practitioners and staff involved with health care service and support. The principle for the health workforce is to work in ways that are responsive, fair and efficient to achieve the best health outcomes possible, given available resources and circumstances.

- **HIPAA:** is an acronym that stands for the Health Insurance Portability and Accountability Act, a US law designed to provide privacy standards to protect patients' medical records and other health information. HIPAA includes information that is provided to health plans, doctors, hospitals and other health care providers. HIPAA has been used as a reference for patient privacy policy worldwide.

- **Host Country** is the term for nations in which organizations or individuals conduct activities such as humanitarian efforts, government intervention or other purposes. While in the host country the guest must comply with the laws and regulations of the host country.
.
- **Host Organizations** is the local entity in the host country that invites and support the sending organization and volunteers. While the role of the host organization will vary based on the specifics of a program, the responsibilities of host organizations typically include; receiving the guests, providing facilities for the program, furnishing in-country resources, and providing local expertise required for the successful implementation of the in-country program.

- **Humanitarian Organizations/NGOs** are charitable entities (termed as 501 C 3 organizations in the US) which provide philanthropic programs and support to help people in need. The primary purpose of humanitarian NGO organizations are efforts such as; saving lives, reducing suffering and maintaining respect to human dignity and addressing health and social economic situations.

- **Information Technology (IT)** is the development, maintenance and use of computers, storage, networking and other physical devices. This includes the infrastructure and processes to create, process, store, secure and exchange all forms of electronic data - for the purpose of processing and distributing data and information.

- **In-country NGOs (INGO)** – are humanitarian charitable organizations within the host country that create and manage philanthropic programs to support and serve people in need. The INGOs are major partners for medical missions - along with other groups such as churches, school, government agencies, universities etc.

- **Laboratory (medical)** is a facility established to conduct tests in order to obtain information about the health of a patient pertaining to diagnosis, treatment, and prevention of disease. Clinical laboratories are focused on applied science, as opposed to research laboratories that focus on basic science.

- **Medical Missions**: Medical Missions are teams of individuals, generally including healthcare professionals (doctors, nurses, and other medical professionals) and support individuals traveling to a LMIC country to provide medical services, and training to help support the health of the population.

Medical Mission are often classified in three time durations:

- Short-term missions - 4 weeks of less
- Medium-term missions - Five weeks to 6 months
- Long-term missions - 7 months to 2 years

- **Medical Products, Vaccines and Technologies** are elements essential for a well-functioning health system. To be effective, the products, vaccines and technologies must be of assured quality, safety, efficacy and cost-effectiveness and appropriate for local needs.

- **Ministry of Health** (MoH) is the national government department responsible for issues related to the general health of the citizenry. Health departments also compile statistics about health issues. The head of the Ministry of Health is often called the Minister of Health.

- **Mission Statement** is a written declaration of an organization's core purpose and focus. Mission statements normally remain unchanged over time. Mission statements serve as 1) filters to separate what is important to the organization from what is not 2) clearly state which area will be served and how 3) communicate a sense of intended direction to the entire organization.

- **Monitor and Evaluation** (**M&E**) is a process designed to helps improve performance and achieve results. Monitoring is the systematic and routine collection of information during a program to track and account for resources used to help make decisions on program execution. Evaluation is the assessment of a project after completion, to review and determine the relevance, effectiveness, efficiency, impact and sustainability of the program.

- **Office of Foreign Assets Control** (**OFAC)** is the financial intelligence and enforcement agency of the US Treasury Department charged with the planning and execution of economic and trade sanctions in support of U.S national security and foreign policy objective. OFAC carries out its activities against foreign states as well as a variety of problematic organizations and individuals, like terrorist groups, deemed to be a threat to U.S. national security **Minister of Foreign Affairs** is a Canadian governmental department that conduct similar function for Canada

- **Paternalistic Attitude** is behavior by an individual, organization or state that limits the liberty or autonomy of others for what is presumed to be for "their own good". Paternalism can imply behavior taken regardless of the will of others and expressed in an attitude of superiority. It is usually unwelcomed as it comes with control and condescension

- **Patient Safety** is a fundamental principle of heath care where actions are taken to protect the patient from harm. Patient Safety is a major WHO directive. Patient safety includes a wide range of actions including infection control, safe use of medicines, equipment safety, safe clinical practice and safe environment of care.

- **Partnership of Quality Medical Donations (PQMD**) is a global alliance of corporations and nongovernmental organizations. PQMD is leading the development and championing of high standards in medical supply and service donations. PQMD seeks

to enhance access to health care in underserved communities and in areas affected by disasters.

- **PQMD Healthcare System Strengthening /Medical Mission Initiative (PQMD HSS/MM)** is a major 3-year effort to explore the shortcoming of current practices in medical mission and work to design and develop guidelines that better connect medical mission volunteers with the needs and priorities of national healthcare systems. In addition, PQMD HSS/MM aims to create a coalition of organizations, experts, institutions and individuals that will be a consultant group to continuously update information, share knowledge and innovation and keep guidance relevance and valuable to all those involved.

- **Primary Care** is the day-to-day healthcare given by a healthcare provider-typically the first point of contact for the patient within the healthcare system. Patients commonly receive primary care from professionals such as a physician or physician assistant. In some localities the primary care professional may be a nurse, pharmacist, or a clinical officer (as in parts of Africa), Depending on the nature of the health condition, patients may then be referred for secondary or tertiary care facilities.

- **Safe Surgery** is the initiative by the World Health Organization (WHO) to improve the safety of surgical care around the world by defining a core set of safety standards that could be applied in all WHO Member States. Safe Surgery publications include the 2009 WHO "Safe Surgery Saves Lives" directive and the 2008 WHO safe surgery Checklist

- **Surgery** is the treatment of injuries or diseases by cutting open the body and removing the damaged part. Surgical procedures are commonly categorized by urgency, type of procedure, body system involved, degree of invasiveness, and special instrumentation. Examples of surgical specialties include; cardiothoracic, colon and rectal, general surgery, gynecology and obstetrics, gynecologic oncology, neurological, ophthalmic, oral and maxillofacial, orthopedic surgery, otorhinolaryngology, pediatric, plastic, urology, and vascular surgery.

- **Secondary Care** is medical care provided by a specialist or facility upon referral from the primary care provider. Secondary health care providers include cardiologists, urologists, dermatologists and other such specialists. The health care services received in secondary care facilities include acute care, short period stay in a hospital emergency department for brief but serious illness.
  Standards are rules or principles used as a basis for judgement and may include authoritative statements that articulate acceptable levels of performance.

- **Sending Organizations (medical missions)** are groups that send out medical mission or medical brigades to LMIC countries. Examples of medical mission sending organizations are; Humanitarian organizations, Faith-based NGOs, Colleges/Universities/Medical schools. Churches/religious organizations etc. Ideally, sending organizations are responsible for screening of volunteers, providing orientation and preparation of volunteers for the local program, arranging for the travel and safety of the volunteer, planning for the ethical and successful implementation of humanitarian health program – which will be conducted in partnership with the host organizations in-country.

- **SPHERE Project** is a voluntary association of various humanitarian organizations designed to set standards to improve the quality of assistance provided to people affected by disasters The SPHERE project also sets standards to enhance the accountability of the humanitarian system in disaster response. The primary publication of the SPHERE project is the handbook, *Humanitarian Charter and Minimum Standards in Disaster Response.* Members of the SPHERE project includes organizations such as Care International, Caritas, International Federation of the Red Cross and Red Crescent, Lutheran World Relief, Oxfam, The World Council of Churches, and Medescins Sans Frontieres (Doctors without Borders).

- **Tropical Health Education Trust (THET)** is the UK Health support organization that provides links between health institutions in Africa, Asia and elsewhere in the world and their counterparts in the UK. THET identifies health priorities then links them with a health institution in the UK that has the knowledge and skills to help them address their health priorities. THET also provides advice and support, such as accessing funding, evaluation and connections with Government agencies.

- **Tertiary Care** is the highly specialized medical care and healthcare facility that provides medical service over an extended period of time. The patients are referred to tertiary care from primary or secondary health professionals. Tertiary health care is provided in a facility that has facilities for advanced medical investigation and treatment. Tertiary care involves advanced and complex procedures and treatments performed by medical specialists. Services provided include care such as cancer management, neurosurgery, cardiac surgery and a host of complex medical and surgical interventions.

- **World Health Organizations** (WHO) is the specialized agency of the United Nations that is concerned with global health. The WHO currently defines its role in public health as follows:

    - providing leadership on matters critical to health
    - shaping the health research and disseminating valuable knowledge;
    - setting norms and standards and promoting and monitoring their implementation;
    - articulating ethical and evidence-based policies;
    - providing technical support, catalyzing change, and building sustainable institutional capacity;
    - monitoring the health situation and assessing health trends.
