# Supplementary material for: Guidelines for short-term medical missions: perspectives from host countries
Source: Global Health. 2022 Feb 19;18:19. doi: 10.1186/s12992-022-00815-7 (PMC8857875; doi:10.1186/s12992-022-00815-7)
Supplement: Supplementary file 2 — Additional file 2. PQMD-HSS Medical Mission Guidelines. [file 12992_2022_815_MOESM2_ESM.docx]

Additional file 2

List of working group members and organizations

Dr. Julie Varughese, Dr. Anne Peterson, Randy Weiss and Julie Winn, Americares; Dr. Timothy Amukele, Pathologists Overseas & Johns Hopkins University; Veronica Arroyave, Baxter Foundation; Darnelle Bernier, Catholic Medical Mission Board; Doug Fountain, Christian Connections for International Health; Judy Hastert and Carla Orner, Heart to Heart International; Samuel Ingram, Medtronic; Kim Keller, Johnson & Johnson; Elizabeth Ashbourne, Juliemarie Vander Burg and Myron Aldrink, Partnership for Quality Medical Donations; Dr. Patti Tracey and Trent University;

David Obando Venegas, Trent Fleming School of Nursing, Trent University, Canada and Honduras; Dr. Philip Wendschuh, North Ohio Heart Ohio Medical Group; Dr. Sarah Brown, Pathologists Overseas & Washington University; Wade Jones, Medtronic; Claudia Sighomnou, HPIC Canada
