# Supplementary material for: Guidelines for short-term medical missions: perspectives from host countries
Source: Global Health. 2022 Feb 19;18:19. doi: 10.1186/s12992-022-00815-7 (PMC8857875; doi:10.1186/s12992-022-00815-7)
Supplement: Supplementary file 3 — Additional file 3. PQMD-HSS Host and Sending Country Checklists. [file 12992_2022_815_MOESM3_ESM.pdf]

Short Term Medical Missions Checklist – Host Organizations

Pre-Departure

- ☐ **Resource Assessment**
  - Have local resources available for the STMM [ie. facilities, transportation, infrastructure, & local Health Care Providers (HCPs)]
  - Have all products & supplies including medicines and technology been identified?
  - Is this STMM the best use of potentially limited human resources within the local system?
  - What is the expected size of the targeted community for the STMM?
- ☐ **Cost/Benefit Assessment**
  - Has the impact of this STMM on local staff/community been assessed?
  - Does the purpose of this STMM align with the Ministry of Health's strategic priorities?
  - Does the STMM fill a gap in the current health care system?
- ☐ **Local Culture Assessment**
  - Does the sending organization have an awareness of the local population, culture and health care system?
- ☐ **Partnership/Alliance**
  - Have the goals of the STMM been identified (including the program expectations of the targeted patient care, health benefits and other activities such as training?
  - Have the terms of the partnership with the sending organization been defined and documented (duration/schedule, use of in-country finances or resources, roles/responsibilities)?
  - Has the sending organization been informed of any past or ongoing partnerships for potential collaboration with other organizations?
- ☐ **Governance**
  - Has proof of due diligence and governance been provided by both organizations through proper documentation?
  - Has the sending organization been provided with relevant health laws and practices?
  - Has the sending organization been provided with local ethical practices or concerns to comply with?
  - Has the scope of practice for STMM participants been defined?
- ☐ **Code of Conduct**
  - Has a code of conduct been defined for the local/national staff involved in the STMM?
  - Has the expected behaviour of the sending organization been defined in a letter of intent (should be informed by local practices, social norms, religious considerations and general cultural awareness)?

- **Preparation**
  - Has a community immersion/orientation been prepared for the sending organization?
  - Has the proper legal authorization been conducted for the importation of donated medicines and medical practice by foreign health personnel?
  - Has the local community/team been prepared for the arrival of the STMM (ie potential outreach to remote communities or specific resources/medicines/training available from the STMM)?

<u>In-Country</u>

- **Implementation**
  - Has ongoing feedback regarding the STMM been provided to the sending organization?
  - Have regular meetings been scheduled to maintain clear communication about roles and responsibilities throughout the STMM?
  - Have efforts been made by the sending organization to build off local facility capacity with the goal of continuity of care?
  - Has a referral system (locally & remote) been established to follow-up patients seen during the STMM?
  - Has proper reporting and filing of patient information been ensured (privacy of health information and consent for treatment)?
  - **Training/Capacity Building**
  - Have opportunities to obtain training for local health staff been sought out (based on what is culturally appropriate/locally attainable? (*consider pre departure; implementation in country)
  - Have key local officials/community members been informed of training development?
  - Have efforts to obtain local funds for training/local program capacity building been conducted (with the goal to best integrate the program into the local health care system)?

<u>Post-Mission Follow Up</u>

- **Sustainability**
  - Have attempts to establish joint efforts for long-term sustainability been conducted?
  - Have local preventative measures been considered within the community (shift from curative treatment to preventative care)?
- **Monitoring and Evaluation**
  - Have appropriate outputs from STMM been identified and obtained (ie impact on patients, impact on training, number of individuals trained)?
  - Has the sending organization been provided with relevant data, information and outputs based on the terms of the partnership?
  - Has the sending organization received a debrief of the STMM effort from the host organizations perspective?
  - Has a final report been drafted on how the STMM contributes to the goal of creating locally sustainable health programs?

Lista de verificación de misiones médicas a corto plazo - Organizaciones anfitrionas

__Antes de la partida__

Evaluación de recursos

- Tenga recursos locales disponibles para el STMM [es decir. instalaciones, transporte, infraestructura y proveedores locales de atención médica (HCP)]

- ¿Se han identificado todos los productos y suministros, incluidos los medicamentos y la tecnología?

- ¿Es este STMM el mejor uso de recursos humanos potencialmente limitados dentro del sistema local?

- ¿Cuál es el tamaño esperado de la comunidad objetivo para el STMM?

Evaluación de costo / beneficio

- ¿Se ha evaluado el impacto de este STMM en el personal / comunidad local?

- ¿El propósito de este STMM se alinea con las prioridades estratégicas del Ministerio de Salud?

- ¿El STMM llena un vacío en el sistema de salud actual?

Evaluación de cultura local

- ¿La organización emisora tiene conocimiento de la población local, la cultura y el sistema de atención médica?

Asociación / Alianza

- ¿Se han identificado los objetivos del STMM (incluidas las expectativas del programa de atención al paciente, beneficios de salud y otras actividades como la capacitación)?

- ¿Tener los términos de la asociación con la organización de envío ha sido definido y documentado (duración / cronograma, uso de recursos o finanzas del país, roles / responsabilidades)?

¿Se ha informado a la organización de envío de alguna asociación pasada o en curso para una posible colaboración con otras organizaciones?

Gobierno

- ¿Han proporcionado ambas organizaciones prueba de la debida diligencia y gobernanza mediante la documentación adecuada?

- ¿Se ha proporcionado a la organización de envío las leyes y prácticas de salud relevantes?

- ¿Se le ha proporcionado a la organización de envío prácticas éticas locales o preocupaciones que cumplir?

- ¿Se ha definido el alcance de la práctica para los participantes de STMM?

Código de conducta

- ¿Se ha definido un código de conducta para el personal local / nacional involucrado en el STMM?

- ¿Se ha definido el comportamiento esperado de la organización de envío en una carta de intención (debe ser informada por las prácticas locales, las normas sociales, las consideraciones religiosas y la conciencia cultural general)?

Preparación

- ¿Se ha preparado una inmersión / orientación comunitaria para la organización de envío?

- ¿Se ha llevado a cabo la autorización legal adecuada para la importación de medicamentos y prácticas médicas donadas por personal de salud extranjero?

- ¿Se ha preparado la comunidad / equipo local para la llegada del STMM (es decir, alcance potencial a comunidades remotas o recursos / medicamentos / capacitación específicos disponibles del STMM)?

<u>**En el país**</u>

Implementación

- ¿Se han proporcionado comentarios continuos sobre el STMM a la organización de envío?

- ¿Se han programado reuniones periódicas para mantener una comunicación clara sobre roles y responsabilidades en todo el STMM?

- ¿La organización de envío ha hecho esfuerzos para desarrollar la capacidad de las instalaciones locales con el objetivo de la continuidad de la atención?

- ¿Se ha establecido un sistema de referencia (local y remoto) para el seguimiento de pacientes atendidos durante el STMM?

- ¿Se ha asegurado la notificación y el archivo adecuados de la información del paciente (privacidad de la información de salud y consentimiento para el tratamiento)?

Capacitación / desarrollo de capacidades

¿Se han buscado oportunidades para obtener capacitación para el personal de salud local (basado en lo que es culturalmente apropiado / localmente alcanzable? (* Considere antes de la partida; implementación en el país)

- ¿Se ha informado a los funcionarios locales clave / miembros de la comunidad sobre el desarrollo de la capacitación?

- ¿Se han realizado esfuerzos para obtener fondos locales para capacitación / desarrollo de capacidades del programa local (con el objetivo de integrar mejor el programa en el sistema local de atención médica)?

<u>Seguimiento posterior a la misión</u>

Sostenibilidad

- ¿Se han realizado intentos de establecer esfuerzos conjuntos para la sostenibilidad a largo plazo?

- ¿Se han considerado medidas preventivas locales dentro de la comunidad (pasar del tratamiento curativo a la atención preventiva)?

Monitoreo y evaluación

- ¿Se han identificado y obtenido resultados apropiados de STMM (es decir, impacto en los pacientes, impacto en la capacitación, número de personas capacitadas)?

- ¿Se ha proporcionado a la organización de envío datos, información y resultados relevantes basados en los términos de la asociación?

- ¿Ha recibido la organización emisora un informe del esfuerzo de STMM desde la perspectiva de las organizaciones anfitrionas?

- ¿Se ha redactado un informe final sobre cómo el STMM contribuye al objetivo de crear programas de salud localmente sostenibles?

Short Term Medical Missions Checklist – Sending Organization

Pre-Departure

- ☐ **Needs assessment** of population or community
    - Does the proposed mission's objectives align with the host country's Ministry of Health's strategic plan?
    - Does it fill an identified gap?
- ☐ **Resource assessment**
    - Programmatic costs ex. for training/capacity building, cost of health services, or available medical personnel
    - Logistical costs for volunteers (travel/accommodation, translators, safety/security)
- ☐ **Cultural assessment**
    - Relevant information about local culture/health systems obtained
- ☐ **Partnership**
    - Does the sending organization have partnerships with local health care providers?
    - Are both partners in agreement on the objectives of the STMM?
    - Are the terms of partnership clearly defined & documented? Including on-going follow up
- ☐ **Governance**
    - Has appropriate due-diligence been conducted for each in-country partner?
    - Has proper legal authorization (permits/licensing) by the host country been granted?
    - Only qualified/licensed Health Care Providers (HCPs) should provide medical/surgical care – has scope of practice clearly been defined for HCPs? Are there measures in place to ensure participants work within their scope of practice?
- ☐ **Code of Conduct**
    - Has a code of conduct been established for volunteers/local partners/STMM staff?
    - Has a letter of intent ex. Memorandum of Understanding (MOU) for all parties been developed/updated?
- ☐ **Preparation**
    - Necessary resources/products have been provisioned
    - STMM staff have been trained on local health laws/practices, safety/security information, relevant cultural sensitivities
    - All staff have necessary immunizations for travel
    - All travel logistics/emergency contingencies have been coordinated

In-Country

- ☐ **Implementation**
  - Regular meetings with in country partners to maintain clear communication and responsibilities during STMM
  - Does the STMM build off local medical standards/capacities
  - Proper reporting/filing of patient information – ensuring consent and privacy maintained
  - All students being appropriately supervised/working within scope of practice
  - Continuity of care – has a referral system for follow up been established (if required)? Has the potential for long-term in-country program implementation been discussed?
- ☐ **Training/Capacity Building**
  - Have opportunities to train/build capacity for local health staff been sought out?
  - Has health training been coordinated with local workforce and officials?
  - Has the opportunity to provide initial resources for training/competency development been assessed (with the goal of integrating any program into the local health system to avoid dependency)?

Post-Mission Follow Up

- ☐ **Sustainability**
  - Have attempts to establish efforts for long-term sustainability been conducted?
  - Focus on prevention and development efforts, not "treat and leave"
  - Potential continuity with local partner for on-going trips or in-country based programs by helping facilitate collaboration with local partners
  - Has the role of providing remote follow-up or guidance for patient care been established with clarity of ongoing roles/responsibilities?
- ☐ **Monitoring and Evaluations**
  - Have metrics to evaluate the current STMM, as well as future efforts and program improvements, been developed relative to in-country health metrics?
  - Have short, medium and long-term measures of success been established for reporting on follow-up and ongoing care?
  - Have appropriate outputs from STMM been identified and obtained (ie funds spent, number of volunteers, number of patients served)?
  - Has a final report on collaborative future plans/ongoing needs been drafted?

PQMD
Partnership for Quality Medical Donations

Lista de verificación de misiones médicas a corto plazo - Organización de envío

__Antes de la partida__

Evaluación de necesidades de población o comunidad

- ¿Los objetivos de la misión propuesta se alinean con el plan estratégico del Ministerio de Salud del país anfitrión?

- ¿Llena un vacío identificado?

Evaluación de recursos

- Costos programáticos ex. para capacitación / desarrollo de capacidades, costo de servicios de salud o personal médico disponible

- Costos logísticos para voluntarios (viajes / alojamiento, traductores, seguridad / protección)

Evaluación cultural

- Información relevante sobre cultura local / sistemas de salud obtenidos

Asociación

- ¿La organización de envío tiene asociaciones con proveedores locales de atención médica?

- ¿Ambos socios están de acuerdo con los objetivos del STMM?

- ¿Están los términos de la asociación claramente definidos y documentados? Incluyendo seguimiento continuo

Gobierno

- ¿Se ha llevado a cabo la debida diligencia adecuada para cada socio en el país?

- ¿Se ha otorgado la autorización legal adecuada (permisos / licencias) por parte del país anfitrión?

Solo los proveedores de atención médica (HCP) calificados / con licencia deben proporcionar atención médica / quirúrgica: ¿se ha definido claramente el alcance de la práctica para los HCP? ¿Existen medidas para garantizar que los participantes trabajen dentro de su ámbito de práctica?

Código de conducta

- ¿Se ha establecido un código de conducta para voluntarios / socios locales / personal de STMM?

- Tiene una carta de intenciones

Memorando de Entendimiento (MOU) para todas las partes ha sido desarrollado / actualizado?

Preparación

- Se han aprovisionado los recursos / productos necesarios

- El personal de STMM ha sido capacitado en leyes / prácticas locales de salud, información de seguridad / protección, sensibilidades culturales relevantes

- Todo el personal tiene las vacunas necesarias para viajar.

- Toda la logística de viajes / contingencias de emergencia ha sido coordinada

En el pais

Implementación

Reuniones periódicas con socios en los países para mantener una comunicación clara y responsabilidades durante el STMM

¿El STMM se basa en estándares / capacidades médicas locales?

Informe / archivo adecuado de la información del paciente: se garantiza el consentimiento y la privacidad.

Todos los estudiantes están adecuadamente supervisados / trabajando dentro del alcance de la práctica.

Continuidad de la atención: ¿se ha establecido un sistema de referencia para el seguimiento (si es necesario)? ¿Se ha discutido el potencial para la implementación de programas a largo plazo en el país?

Capacitación / desarrollo de capacidades

¿Se han buscado oportunidades para capacitar / desarrollar capacidades para el personal de salud local?

¿Se ha coordinado la capacitación en salud con la fuerza laboral y los funcionarios locales?

¿Se ha evaluado la oportunidad de proporcionar recursos iniciales para el desarrollo de capacitación / competencia (con el objetivo de integrar cualquier programa en el sistema de salud local para evitar la dependencia)?

Seguimiento posterior a la misión

Sostenibilidad

¿Se han realizado intentos de establecer esfuerzos para la sostenibilidad a largo plazo?

Centrarse en los esfuerzos de prevención y desarrollo, no "tratar y salir"

Posible continuidad con el socio local para viajes en curso o programas basados en el país al ayudar a facilitar la colaboración con los socios locales.

¿Se ha establecido la función de proporcionar seguimiento u orientación remotos para la atención al paciente con claridad de las funciones / responsabilidades actuales?

Monitoreo y evaluaciones

¿Se han desarrollado métricas para evaluar el STMM actual, así como esfuerzos futuros y mejoras del programa, en relación con las métricas de salud en el país?

¿Se han establecido medidas de éxito a corto, mediano y largo plazo para informar sobre el seguimiento y la atención continua?

¿Se han identificado y obtenido los resultados apropiados de STMM (es decir, fondos gastados, número de voluntarios, número de pacientes atendidos)?

¿Se ha elaborado un informe final sobre planes futuros de colaboración / necesidades actuales?
